# Systemic Effects of Hemorrhagic Snake Venom Metalloproteinases: Untargeted Peptidomics to Explore the Pathodegradome of Plasma Proteins

**DOI:** 10.3390/toxins13110764

**Published:** 2021-10-28

**Authors:** Luciana Bertholim, Alison F. A. Chaves, Ana K. Oliveira, Milene C. Menezes, Amanda F. Asega, Alexandre K. Tashima, Andre Zelanis, Solange M. T. Serrano

**Affiliations:** 1 Laboratório de Toxinologia Aplicada, Center of Toxins, Immune-Response and Cell Signalig, CeTICS, Instituto Butantan, São Paulo 05503-900, SP, Brazil; luciana.nasciben@gmail.com (L.B.); alison.chaves@butantan.gov.br (A.F.A.C.); ankaoliv@gmail.com (A.K.O.); menezes.milene@gmail.com (M.C.M.); amanda.asega@gmail.com (A.F.A.); 2 Department of Biochemistry, Escola Paulista de Medicina, Federal University of Sao Paulo, Sao Paulo 04023-901, SP, Brazil; aktashima@unifesp.br; 3 Functional Proteomics Laboratory, Department of Science and Technology, Federal University of São Paulo (UNIFESP), 330 Talim St., São José dos Campos 12231-280, SP, Brazil; andre.zelanis@unifesp.br

**Keywords:** *Bothrops jararaca*, HF3, human plasma, proteolysis, snake venom metalloproteinase

## Abstract

Hemorrhage induced by snake venom metalloproteinases (SVMPs) is a complex phenomenon that involves capillary disruption and blood extravasation. HF3 (hemorrhagic factor 3) is an extremely hemorrhagic SVMP of *Bothrops jararaca* venom. Studies using proteomic approaches revealed targets of HF3 among intracellular and extracellular proteins. However, the role of the cleavage of plasma proteins in the context of the hemorrhage remains not fully understood. The main goal of this study was to analyze the degradome of HF3 in human plasma. For this purpose, approaches for the depletion of the most abundant proteins, and for the enrichment of low abundant proteins of human plasma, were used to minimize the dynamic range of protein concentration, in order to assess the proteolytic activity of HF3 on a wide spectrum of proteins, and to detect the degradation products using mass spectrometry-based untargeted peptidomics. The results revealed the hydrolysis products generated by HF3 and allowed the identification of cleavage sites. A total of 61 plasma proteins were identified as cleaved by HF3. Some of these proteins corroborate previous studies, and others are new HF3 targets, including proteins of the coagulation cascade, of the complement system, proteins acting on the modulation of inflammation, and plasma proteinase inhibitors. Overall, the data indicate that HF3 escapes inhibition and sculpts the plasma proteome by degrading key proteins and generating peptides that may act synergistically in the hemorrhagic process.

## 1. Introduction

Among the drastic consequences of viperid snakebite envenomation, manifestations of local tissue damage, such as hemorrhage and myonecrosis, may result in permanent tissue damage and sequelae [1,2]. In cases of severe envenomation, bleeding in organs distant from the site of bite, such as the heart, lungs, kidneys, and brain, may also occur [3,4,5]. Coagulopathy, including procoagulant, blood-clotting, fibrinolytic, and anticoagulant effects, is another cause of morbidity and mortality upon viperid snakebite accidents [6,7]. Local and systemic effects of viperid envenomation involve the synergistic effects of snake venom metalloproteinases (SVMPs) on plasma proteins, connective tissue, platelets, and blood vessels. SVMPs are zinc-dependent enzymes classified in the M12B subfamily of metallopeptidases, in which the P-III class protein precursors are comprised pro-, catalytic, disintegrin-like, and cysteine-rich domains [8]. SVMPs target specific capillary basement components, cell surface proteins, and extracellular matrix and plasma proteins, thereby promoting capillary rupture and content extravasation, and resulting in hemostasis disturbance and hemorrhage [8,9,10,11].

HF3 is a very potent P-III class SVMP of *Bothrops jararaca* venom that induces local hemorrhage with minimum hemorrhagic doses of 15 ng on rabbit skin, and 160 ng on mouse skin [12,13]. The precursor of HF3 is composed of 606 amino acid residues, including five putative *N*-glycosylation sites. The calculated molecular mass of the mature form of HF3 is 46 kDa, whereas on SDS-PAGE, it shows a mobility corresponding to a protein of ~70 kDa, indicating that it is heavily glycosylated [13,14]. HF3 was shown to degrade proteins of the plasma and extracellular matrix, including fibrinogen, fibronectin, vitronectin, von Willebrand factor, collagens IV and VI, laminin, matrigel, antithrombin III, complement components C3 and C4, prothrombin, and plasminogen in vitro [9,15]. Moreover, HF3 showed degradation or limited proteolysis of the proteoglycans aggrecan, brevican, biglycan, decorin, glypican-1, lumican, mimecan, and syndecan-1 [15]. Proteins extracted from the hemorrhagic dorsal skin of mice injected with HF3 were submitted to SDS-PAGE and immunostained with specific anti-proteoglycan antibodies, resulting in the demonstration of in vivo cleavage of biglycan, decorin, glypican-1, lumican, and syndecan-1 [15]. Interestingly, HF3 cleaved the platelet derived growth factor receptor (PDGFR; alpha and beta), and PDGF, in vitro, and both receptor forms were also detected as degraded in vivo in the hemorrhagic process generated by HF3 in the mouse skin [15]. Moreover, the proteolytic activity of HF3 is not affected by plasma proteinase inhibitors, including α2-macroglobulin, which is cleaved by HF3 [16]. The disintegrin-like and cysteine-rich domains of HF3 play a role in its activities upon cells, as reported on their ability to inhibit collagen-induced platelet aggregation, to activate macrophage phagocytosis mediated by αMβ2 integrin, and to induce inflammation by increasing leukocyte rolling in the microcirculation [14,17,18].

The potent hemorrhage generated by HF3 on the mouse skin was analyzed using proteomic approaches, which corroborated the hydrolysis of intracellular, extracellular, and plasma proteins, including some proteoglycans [19]. Indeed, the cleavage of proteoglycans suggested a critical role of the destabilization of the mouse skin integrity in the hemorrhagic process generated by HF3, along with the release of pro-inflammatory fragments acting in the imbalance of tissue homeostasis [15,19]. Despite the strong evidence of the role of proteolysis in the local hemorrhage promoted by SVMPs, the full substrate repertoire of HF3 is unknown. Recently, we reported positional proteomic studies of HF3 cleavage sites in mouse embryonic fibroblast secreted proteins using terminal amine isotopic labeling of substrates (TAILS), which revealed a number of substrates, including proteins of the extracellular matrix and focal adhesions, and the cysteine protease inhibitor cystatin-C [20]. Proteomic identification of cleavage site specificity (PICS) was also used for identifying cleavage sites and sequence preferences in peptides upon incubation with HF3. Two studies using tryptic libraries of proteins from human plasma [21] and from THP-1 monocytic cells [20] revealed a clear preference for leucine at P1′ position and the influence of amino acid sequences adjacent to the scissile bond in the substrate specificity of HF3, similarly to other metalloproteinases from viperid venoms [22].

The aim of this study was to gain new insights into the mechanisms of hemorrhage production by HF3 by expanding the analysis of the substrate repertoire of this SVMP on plasma proteins. To this end, approaches for the depletion of the most abundant proteins and for the enrichment of low abundant proteins of the human plasma were used to minimize the dynamic range of protein concentration. In order to assess the proteolytic activity of HF3 on a wide spectrum of proteins, we used untargeted peptidomics to detect the degradation products by mass spectrometry.

## 2. Results

**Figure 1:**
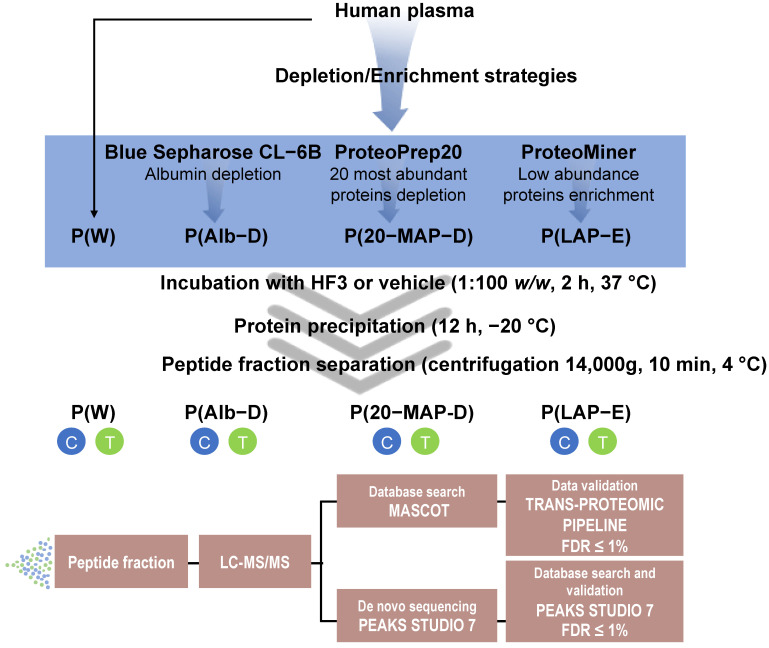
Experimental workflow for the analysis of the proteolytic activity of HF3 on human plasma proteins. Human plasma was depleted of (i) albumin, or (ii) 20 most abundant proteins, or (iii) enriched of low abundant proteins. Whole plasma [P(W)], albumin-depleted plasma [P(Alb-D)], plasma depleted of the 20 most abundant proteins [P(20-MAP-D)], and plasma enriched of low abundant proteins [P(LAP-E)] were individually incubated with HF3, or vehicle, for 2 h at 37 °C. Peptide fractions from control (C) or HF3-treated (T) samples were obtained and analyzed by LC-MS/MS, and peptides were identified using Mascot in conjunction with the TPP and PEAKS Studio.

The aim of this study was to evaluate the proteolytic activity of HF3 on human plasma proteins. In order to overcome the large protein dynamic range and complexity of human plasma [23,24], four different types of human plasma preparations were compared in in vitro incubations with HF3: whole plasma [P(W)]; plasma depleted of albumin [P(Alb-D)]; plasma depleted of 20 most abundant proteins [P(20-MAP-D)]; and plasma enriched of low-abundance proteins [P(LAP-E)]. Further, the resulting degradation products present in the peptide fraction were analyzed by LC-MS/MS and a database search using tools of Mascot in conjunction with the Trans-Proteomic Pipeline (TPP) and PEAKS Studio. An overview of the study is provided in Figure 1, as well as the nomenclature used for the different plasma fractionation methods.

Three individual experiments of incubation of plasma proteins with HF3 were carried out with each of the four types of plasma samples, P(W), P(Alb-D), P(20-MAP-D), and P(LAP-E), and the following criteria were used in the untargeted peptidomic analysis to accept a protein as being cleaved by HF3: (i) peptides identified in the control samples were disregarded in the analysis, therefore only proteins that showed peptides identified exclusively in the sample of plasma treated with HF3 were considered; (ii) only proteins identified in at least two experiments were considered.

### 2.1. Electrophoretic Profiles of Plasma Samples Incubated with HF3

**Figure 2:**
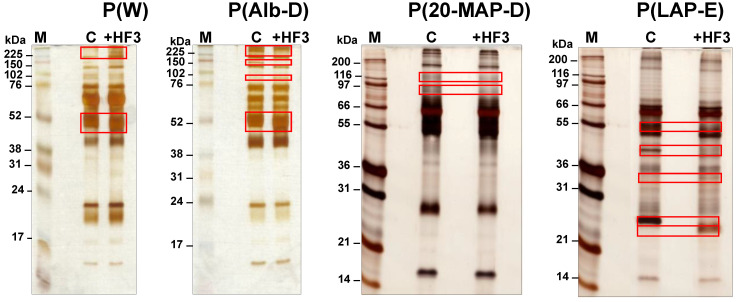
SDS-PAGE profile (12% SDS-polyacrylamide gel) of plasma samples incubated with HF3. M: molecular mass markers; C: control plasma; +HF3: plasma incubated with HF3. Red rectangles indicate regions showing different staining intensities between the control and HF3-treated plasma. Proteins were stained with silver.

As shown in Figure 2, the three methods used for the decomplexation of the plasma proteome to achieve adequate sampling of proteins resulted in distinct electrophoretic profiles, containing proteins of 15−250 kDa. The incubation of these plasma samples with HF3 resulted in protein bands that decreased in intensity in the treated plasma in comparison to the control plasma, indicating proteins that may have been degraded by HF3. The bands that increased in intensity in the treated plasma indicated HF3 proteolysis products derived from proteins of higher molecular mass. In the case of P(W) incubated with HF3, it was possible to observe an increase in the intensity of bands in the region above 225 kDa, and a slight variation in the mobility of an intense band in the region of 52 kDa. P(Alb-D) treated with HF3 showed a decrease in the intensity of bands of ~140 kDa and above, and also a small alteration in the mobility of the ~52 kDa band. On the other hand, the electrophoretic profile of P(20-MAP-D) treated with HF3 showed only a slight decrease in the intensity of the bands in the region between 80 kDa and 120 kDa. However, upon incubation with HF3, P(LAP-E) showed a clear decrease in the intensity of the bands in of ~55 kDa, 40 kDa, and 25 kDa, and an increase in the intensity of the bands of 34 kDa and 22 kDa.

Overall, all electrophoretic profiles indicated differences between control and treated plasma samples, evidencing the proteolytic effect of HF3 on plasma proteins in vitro. Interestingly, the electrophoretic profile of P(LAP-E) showed the most significant differences between control and treated samples, possibly due to the fact that the method applied for the enrichment of low abundant proteins of the plasma proteome was more efficient in minimizing the dynamic range of protein concentration, thus enabling a better visualization of the degraded proteins.

### 2.2. Peptidomic Profiling of Plasma Samples Incubated with HF3

The peptide fractions of control and treated samples of P(W), P(Alb-D), P(20-MAP-D), and P(LAP-E) were subjected to LC-MS/MS, and analyses of peptide identification and spectra counting were carried out by: (i) a Mascot database search and validation of results using the PeptideProphet and ProteinProphet tools of the TPP platform (the results of this approach were designated as ‘Mascot/TPP’); and (ii) de novo sequencing, a database search, and validation of results by Peaks Studio 7 program (the results of this approach were designated as ‘PEAKS’) (Supplementary Tables S1–S8).

**Figure 3:**
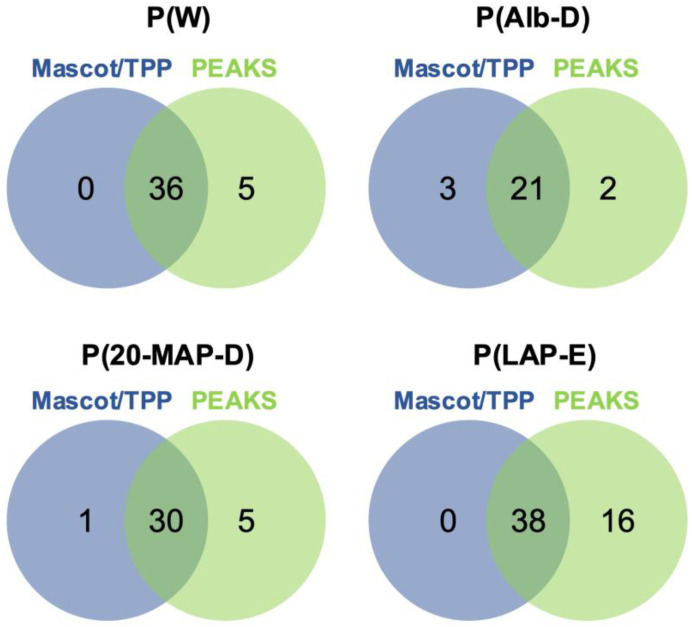
Summary of numbers of plasma proteins identified as degraded by HF3, by LC-MS/MS analysis of the peptide fraction, using four different plasma preparations. Comparison of results obtained using Mascot/TPP and PEAKS Studio 7 for peptide identification.

Figure 3 shows Venn diagrams comparing the total numbers of plasma proteins identified as cleaved by HF3 using both identification approaches. The analysis of whole plasma [P(W)] incubated with HF3 showed 36 proteins as cleaved by HF3 using both identification approaches (Table 1), whereas five proteins were identified exclusively by PEAKS. In the plasma depleted of albumin [P(Alb-D)], we identified 21 proteins cleaved by HF3 using both identification approaches (Table 2), including three proteins exclusively identified by Mascot/TPP, and two proteins exclusively by PEAKS. In the plasma depleted of the 20 most abundant proteins [P(20-MAP-D)], 30 proteins were identified as cleaved by HF3 using both identification approaches (Table 3), with one protein identified exclusively by Mascot/TPP, and five by PEAKS. The incubation of HF3 with the plasma enriched of low-abundance proteins [P(LAP-E)] revealed 38 substrates by both approaches (Table 4), and 16 proteins identified only by PEAKS.

**Figure 4:**
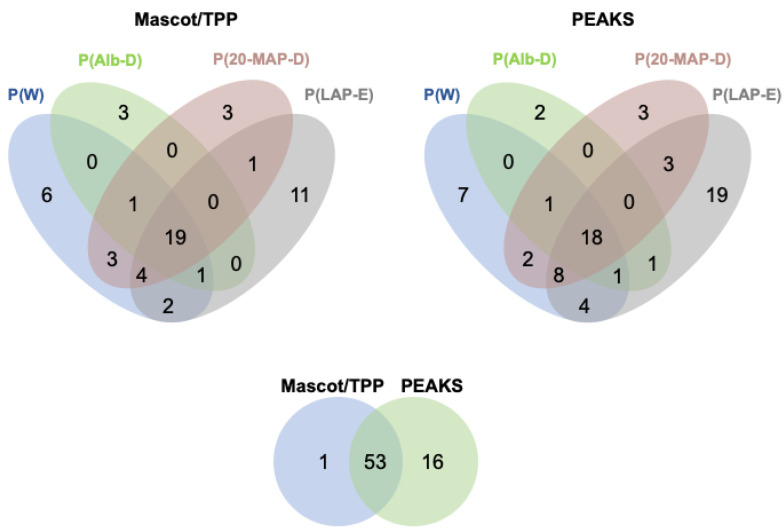
Summary of proteins identified as HF3 substrates. Upper panels: Four-way Venn diagrams of substrate identification using four samples of human plasma, and two methods of protein identification. Lower panel: Summary of proteins identified by each method of protein identification.

Regarding the different methods of depletion of abundant plasma proteins and enrichment of low-abundant proteins, the four-way Venn diagrams displayed in Figure 4 show that the analysis of the peptide fraction of P(LAP-E) provided a higher number of identified HF3 substrates, with 11 substrates indicated by the approach of Mascot/TPP, and 19 substrates indicated by PEAKS. Unexpectedly, the analysis of P(W) incubated with HF3 also provided results on HF3 substrates which were identified exclusively by this method (six substrates by the Mascot/TPP identification approach, and seven substrates by PEAKS), however, it is worth mentioning that the initial amount of proteins used in the incubation of P(W) with HF3 was higher (200 μg) compared to 50 μg for P(Alb-D), P(20-MAP-D), and P(LAP-E). This result can be attributed to the fact that there was less manipulation of the whole plasma sample compared to other approaches, which involved at least one more protein depletion/enrichment step, performed separately (biological replicates), before incubation with the proteinase.

In general, in each method of preparation of human plasma for incubation with HF3, most proteins considered as a target of HF3 for proteolysis were identified by both bioinformatics approaches used, reinforcing the results (Figure 4). Interestingly, both approaches resulted in a higher number of substrates identified in the P(W) and P(LAP-E) plasma samples. Moreover, although the PEAKS approach revealed a higher number of HF3 substrates (70), 53 proteins were identified by both bioinformatics approaches.

### 2.3. Proteins Degraded by HF3 in the Human Plasma

Table 1 shows the list of 41 proteins identified as substrates of HF3 in the P(W) sample. The ten most degraded substrates are proteins involved in functions in the coagulation cascade, complement system, protein transport, and proteinase inhibition.

The identification of substrates of HF3 in the plasma depleted of albumin resulted in only 26 proteins (Table 2). The ten most degraded substrates are proteins involved in functions in the coagulation cascade, complement system, lipid transport, hormone transport, and proteinase inhibition.

In the plasma depleted of the 20 most abundant proteins, 36 proteins were identified as degraded by HF3 (Table 3), whereas in the plasma submitted to enrichment of the low-abundant proteins, 54 proteins were detected as cleaved by HF3 (Table 4). Despite the higher number of substrates identified in the latter, the ten most degraded proteins in both types of plasma preparations were nearly the same. Considering the bioinformatics approaches applied for peptide identification, overall, when using PEAKS, higher numbers of spectra were identified for all degraded proteins.

Overall, a total of 61 proteins (Table 5) were detected as cleaved by HF3, including 18 that were identified in all types of plasma preparations: alpha-2-antiplasmin; alpha-2-HS-glycoprotein; apolipoproteins A-I, A-II, A-IV, C-II, C-III, E, and L1; clusterin; complement C3; fibrinogen alpha and beta chains; inter-alpha-trypsin inhibitor heavy chains H2 and H4; kininogen-1; prothrombin; and transthyretin.

**Figure 5:**
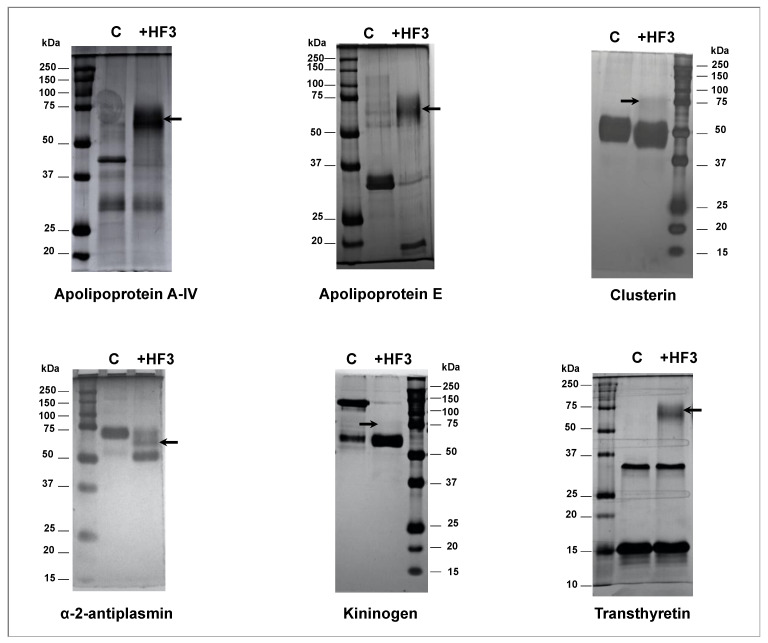
Proteolytic activity of HF3 upon isolated plasma proteins. Apolipoprotein A-IV, apolipoprotein E, clusterin, α-2-antiplasmin, kininogen, and transthyretin were incubated at a 1:10 (*w/w*) enzyme-to-substrate ratio with HF3 for 2 h, as described in Materials and Methods, and subjected to SDS-PAGE. Arrows indicate the band of HF3. Numbers on the right and left indicate molecular mass marker mobility. Proteins were silver stained.

We further investigated the newly identified proteins as HF3 substrates in the human plasma. For this analysis, we selected some proteins that are commercially available and that were detected as cleaved by HF3 using LC-MS/MS analysis. The proteins were incubated at a 1:10 (*w/w*) enzyme-to-substrate ratio with HF3 for 2 h and subjected to SDS-PAGE (Figure 5). Apolipoprotein A-IV was completely degraded by HF3. Apolipoprotein E was almost completely degraded by HF3 to generate a fragment of ~15 kDa. After incubation with HF3, clusterin showed a slight reduction of molecular mass, indicating that HF3 may have promoted its limited proteolysis. HF3 also promoted the limited proteolysis of α-2-antiplasmin, resulting in fragments of ~68 kDa and 55 kDa. The 120 kDa band of high molecular weight kininogen was almost completely degraded by HF3, resulting in a stable fragment of ~68 kDa. In the case of transthyretin, its dimer and monomer bands remained unchanged after incubation with HF3.

**Figure 6:**
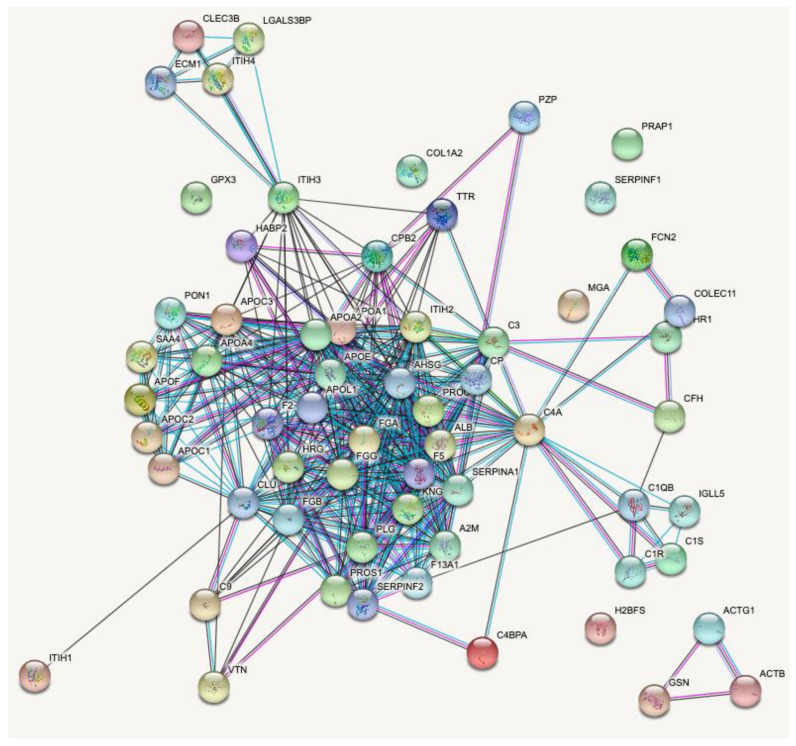
Protein-protein interactions of proteins identified as degraded in human plasma by HF3 according to the STRING database (61 proteins; 401 edges (expected 27); PPI enrichment *p*-value < 1.0^−16^). The connecting lines between protein nodes indicate protein-protein interactions.

Figure 6 shows the protein-protein interaction network of 61 proteins cleaved by HF3 in the human plasma, visualized using STRING analysis [25], evidencing that most of them (53) have connected molecular functions related mainly to the activation and control of the coagulation and complement systems.

### 2.4. Mapping the Primary Specificity of HF3 on Plasma Proteins

**Figure 7:**
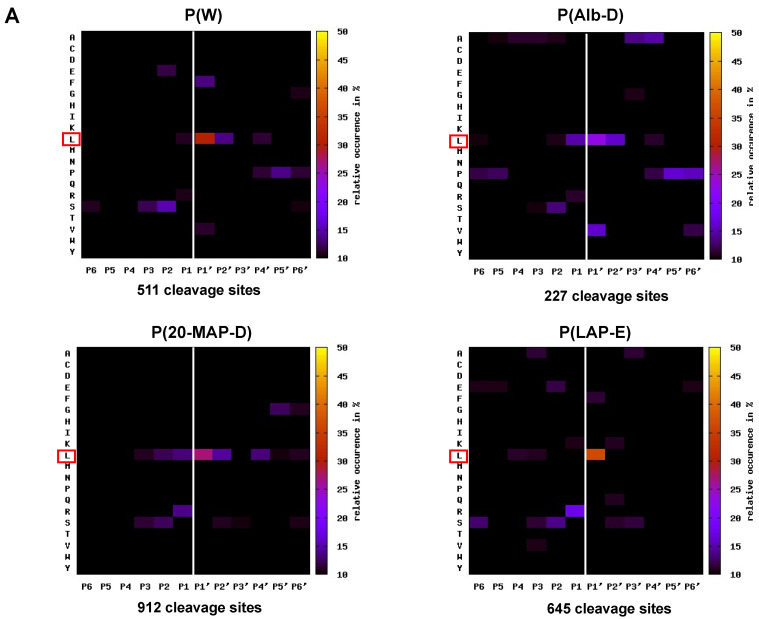

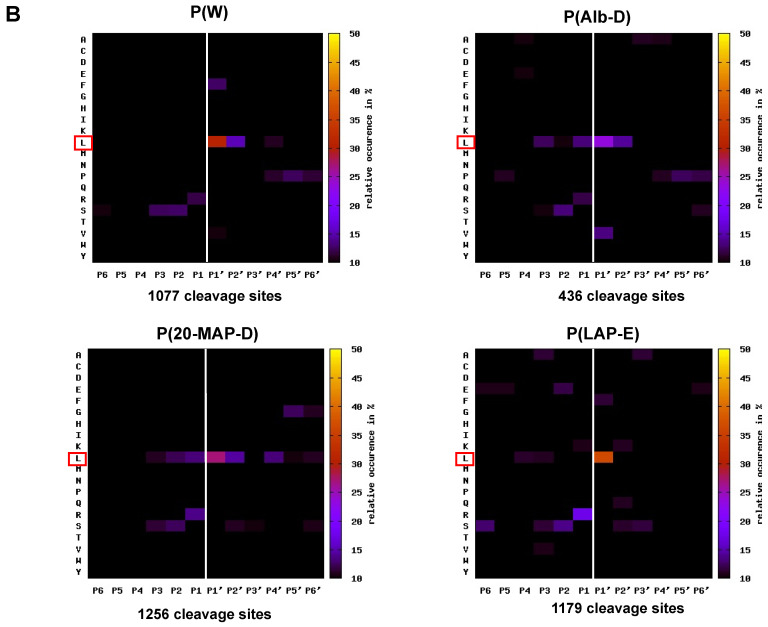
Substrate specificity assessed by the identification of peptides generated by the proteolytic activity of HF3 on human plasma proteins. (**A**) Heat maps showing the relative occurrence (in %) of each amino acid residue and the fold-change over the natural abundance of amino acids, identified using Mascot/TPP. (**B**) Heat maps showing the relative occurrence (in %) of each amino acid residue and the fold-change over the natural abundance of amino acids, identified using PEAKS. Only peptides derived from proteins identified as HF3 substrates were considered, and peptides identified both in control and treated samples were excluded. Heat maps were created at http://clipserve.clip.ubc.ca/pics (accessed on 1 April 2014).

A large number of peptides derived from the activity of HF3 on plasma proteins were identified in the experiments performed with P(W), P(Alb-D), P(20-MAP-D), and P(LAP-E), and these were used for the evaluation of the proteins that were hydrolyzed by HF3, as well as to carry out the mapping of amino acid sequences adjacent to the cleavage sites, which are preferential for the proteinase (primary specificity). For this purpose, we used the amino acid sequences of the peptides, corresponding to the positions P1’–P6’ [26], identified in the samples treated with HF3, and originating from the proteins considered as substrate, whereas the complementary amino acid sequences (P6-P1) adjacent to the cleavage sites of the respective peptides were obtained using a tool available online at http://clipserve.clip.ubc.ca/pics; accessed on 1 April 2014 [27], which also generated the graphical representation (heat map) of the proteinase cleavage consensus sequence. The heat maps representing the occurrence of amino acids at positions P6-P6’ are described in Figure 7, corresponding to the identification results obtained by the Mascot/TPP and PEAKS approaches. It was interesting to verify that, although the number of cleavage sites identified by the PEAKS approach was higher than that of Mascot/TPP, the heat maps generated with data from both approaches from data from all plasma samples were very similar and evidenced a clear preference of HF3 for Leu at the P1’ position.

**Figure 8:**
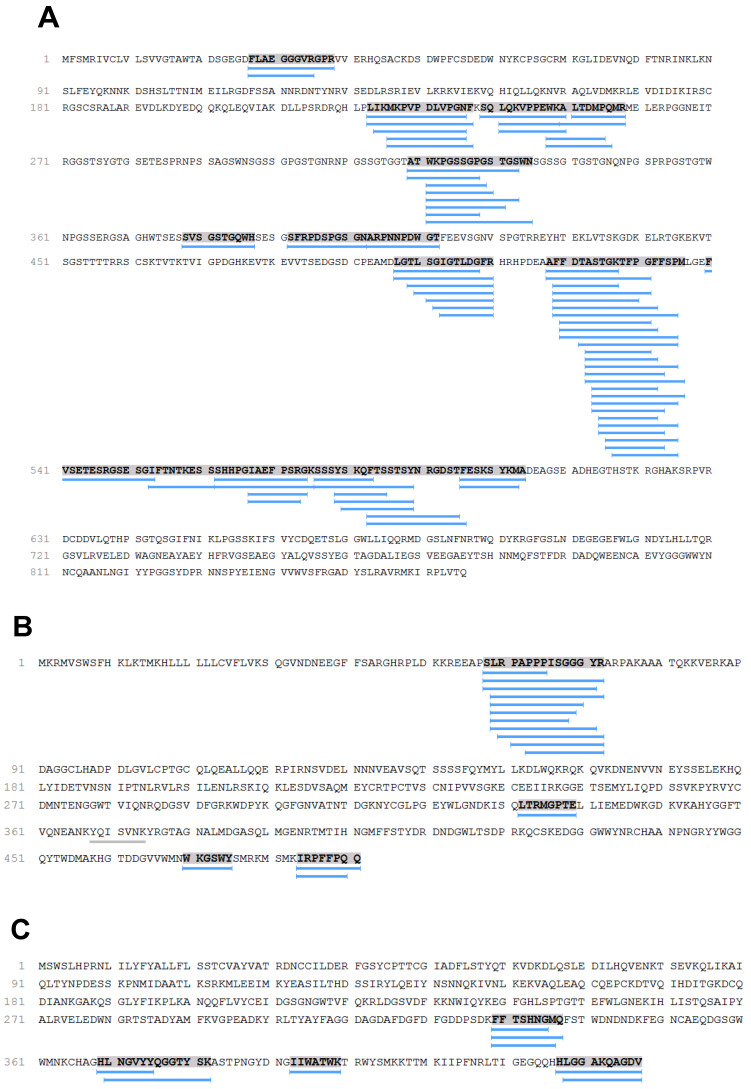
Amino acid sequence of human fibrinogen alpha (**A**), beta (**B**), and gamma (**C**) chains, indicating the identified cleavage products (blue bars) generated by the incubation with HF3. Graphical view generated with PEAKS Studio 7.

The analysis of hydrolysis products of plasma proteins incubated with HF3 showed that fibrinogen was cleaved in the alpha, beta, and gamma chains (Supplementary Material Tables S1–S8). These data corroborate previous studies that described extensive cleavage of the alpha chain, followed by more limited beta chain cleavage [9] by HF3, however, proteolysis of the fibrinogen gamma chain by HF3 was unknown. Here, peptides from the C-terminal region of the fibrinogen gamma chain were identified as a product of HF3 activity in P(20-MAP-D) and P(LAP-E). Studies have shown that the C-terminal region of the fibrinogen gamma chain plays important roles in platelet interaction [28] and fibrin stabilization [29], and that the C-terminal peptide also plays a role in the inhibition of platelet aggregation [30]. Thus, for the validation of peptides generated by cleavage of fibrinogen by HF3 in plasma, it was incubated with isolated fibrinogen, followed by analysis of the resulting peptide fraction by LC-MS/MS. This analysis revealed the peptides generated by the enzymatic activity of HF3, and indirectly, the cleavage points of HF3 in the protein (Supplementary Material Table S9). Figure 8 shows the location of these peptides in the fibrinogen sequence, corresponding to 73 peptides of the alpha chain and 15 peptides of the beta chain. Eight fibrinogen gamma chain peptides were also identified, including two located in the C-terminal region, thus confirming the data obtained by the incubation of HF3 with plasma.

## 3. Discussion

### 3.1. Human Plasma Preparations for Degradomic Analysis

The application of methods for the depletion of the most abundant proteins in human plasma, and for the enrichment of low-abundant proteins, resulted in samples with significantly different electrophoretic profiles, which allowed us to evaluate the proteolytic activity of HF3 on a wide spectrum of proteins in concentrations that allowed the detection of degradation products by mass spectrometry. Regarding the electrophoretic profile, P(LAP-E) showed the clearest differences in comparison to whole plasma, a fact that can be directly attributed to the principle of the ProteoMiner technology, which, by equalizing the proteome of a sample, significantly alters the concentration of proteins poorly represented in a proteome. Millioni et al., 2011, compared techniques for depleting the 20 most abundant proteins with the ProteoPrep 20 column and the enrichment of less abundant proteins with ProteoMiner, for the analysis of the plasma proteome [31]. They reported that the methods were complementary regarding the identified proteins, however, a higher number of proteins was identified in the plasma proteome depleted of the 20 most abundant proteins. Hakimi et al., 2014, tested the efficacy and reproducibility of these methods for plasma protein depletion/enrichment, and by comparing 18 randomly chosen proteins, they found that both techniques were reproducible [32]. However, the protein enrichment approach with ProteoMiner provided a greater number of identified proteins. It is worth emphasizing that a large part of the studies that employ techniques for the depletion of the most abundant proteins in the plasma or enrichment of low-abundant proteins aim to characterize the plasma proteome itself, and more often, the search for biomarkers [33,34,35,36,37]. Studies aimed at analyzing the repertoire of substrates of a proteinase on a given proteome are less frequent, and generally employ methods for the depletion of abundant proteins after the action of the proteinase, in a step prior to one-dimensional or two-dimensional electrophoresis [18,19,38], or do not employ protein depletion/enrichment techniques [39,40,41]. In the present study, plasma protein depletion/enrichment techniques were used in a step prior to incubation with the proteinase. Thus, it was possible to evaluate the proteolytic activity of HF3 through the identification of peptides from the cleaved proteins, for the characterization of the HF3 degradome in the human plasma in vitro.

### 3.2. Analysis of the Plasma Peptide Fraction

Considering that the peptides identified by mass spectrometry after the incubation of plasma with HF3, which were absent in their respective control, are probably products arising from the cleavage of plasma proteins by HF3, the structure of these peptides indicates the substrates degraded by this proteinase. To elucidate the HF3 cleavage products by the analysis of the peptide fraction, we used two bioinformatics approaches with restrictive criteria for peptide identification. In addition, to be considered as HF3 substrate, proteins whose peptides were identified in the peptide fraction were again subjected to restrictive criteria for comparing samples treated with HF3 and control: (i) the peptides identified in the control samples were subtracted from the list of peptides identified in the HF3-treated samples, as they were potentially not generated by HF3, thus, only peptides identified exclusively in the treated sample remained in the analysis; (ii) proteins were considered cleaved by HF3 if their peptides (hydrolysis products) were present in at least two of the three experiments performed. In view of these criteria, we identified that 70 protein entries present in the UniProt database (considering individual oligomeric protein chains as different entries) were identified as HF3 substrates, and of these, 61 are unique proteins. Some of these proteins corroborate data described in the literature and others are considered new substrates of this metalloproteinase.

In general, for each method of preparation of human plasma, a large portion of the proteins considered as a target of HF3 were identified by both bioinformatics approaches used. The PEAKS approach provided a greater number of proteins identified in P(W), P(20-MAP-D), and P(LAP-E), whereas the Mascot/TPP approach provided a greater number of proteins identified as substrate in the P(Alb-D) approach. Zhang et al., 2012, compared, for data from LC-MS/MS analysis of trypsin digestion of *Pseudomonas aeruginosa*, the number of peptide-to-spectrum match (PSM) identified by PEAKS and Mascot, using a database search [42]. The study showed that PEAKS provided 30% more PSMs when compared to Mascot. In the present study, higher numbers of spectral counts and identified peptides were detected in the analysis of the peptide fraction of the plasma incubated with HF3 by PEAKS, nevertheless, most substrates were identified by both bioinformatics approaches.

The direct incubation of some of the new substrate candidates with HF3 and the visualization of cleavage products by SDS-PAGE confirmed its proteolytic activity upon human plasma proteins, as detected by LC-MS/MS analysis. Furthermore, it revealed different outcomes, i.e., limited proteolysis or degradation, resulting from the hydrolysis of plasma proteins by HF3. In the case of transthyretin, however, as most peptides identified as cleavage products derived from the incubation of HF3 with plasma were derived from its N- and C-terminal regions, its direct incubation with HF3 did not show any change in the bands corresponding to its dimer and monomer forms, as visualized by SDS-PAGE.

### 3.3. Analysis of the Primary Specificity of HF3 on Plasma Proteins

The use of peptides generated by the cleavage of plasma proteins by HF3 proved to be an excellent tool to evaluate its primary specificity, since a large number of cleavage sites were analyzed (2295 identified by Mascot/TPP and 3948 by PEAKS) and showed the preference of HF3 for Leu in the P1’ position. In agreement with these results, the analysis of the primary specificity of HF3, using data resulting from cleavage events in native proteins present in the secretome of a mouse embryonic fibroblast analyzed by TAILS technology, showed a predominance of Leu at P1′ position [20]. Interestingly, the analysis of the activity of HF3 on human plasma peptide substrates by the PICS approach showed a similar pattern, in which Leu had a higher occurrence at P1′ position, followed by hydrophobic residues in P2′ [21]. The preference for Leu at the P1′ subsite is a common feature of metalloproteinases, such as matrix metalloproteases (MMPs) [43]. In the study by Kleifeld et al., 2010, the primary specificity of matrix metalloproteinase 2 (MMP-2) was evaluated by the TAILS method, showing a strong preference for Leu at the P1′ position (more than 45% occurrence) and Pro at the P3 position [44]. Using the same approach, Prudova et al., 2010, found that MMP-2 had a strong preference for Leu in position P1′ and Pro in P3, whereas MMP-9 exhibited a more relaxed preference for Leu in P1′ and a very strong preference for Pro in P3, reflecting the differences in the S1′subsites of these proteinases [45]. MMP-10 also revealed its preference for Leu at the P1′ position and Pro at the P3 position [46]. Unlike what has been shown in these studies, the results of the analysis on the primary specificity of HF3 on plasma proteins did not indicate a significant preference for any amino acid at positions other than P1’, indicating that amino acids present at positions adjacent to the cleavage site do not have a relevant role in determining the hydrolysis of peptide bonds in proteins by HF3.

### 3.4. Plasma Proteins Cleaved by HF3

Fibrinogen peptides (alpha and beta chains), vitronectin, and fibronectin were considered as cleaved by HF3, corroborating previous studies by our group that showed that these proteins are hydrolyzed by HF3 in vitro [9]. The results obtained in the present study also confirm data from Paes Leme et al., 2012 [19], who showed that fibronectin and fibrinogen were degraded in the plasma of mice injected in the dorsal skin with HF3. Although fibrinogen alpha and beta chains have already been described as HF3 substrates, in this study, the fibrinogen gamma chain was also detected as a target of this metalloproteinase. Peptides from the fibrinogen gamma chain were identified as a product of HF3 activity on P(20-MAP-D) and P(LAP-E). In addition, incubation of HF3 with isolated fibrinogen, followed by analysis of the resulting peptide fraction by LC-MS/MS, confirmed the presence of these peptides. These analyses revealed the release of peptides from the C-terminal portion of the fibrinogen gamma chain by HF3. In addition to the important role of this region in the stabilization of fibrin by cross-linking [29], studies have shown that the C-terminal region of the gamma chain is involved in its interaction with the αIIbβ3 integrin in platelets [28,47,48]. Farrell et al., 1992, also found that a recombinant fibrinogen, which contained an interruption in the C-terminal region of the gamma chain, lost much of its ability to mediate platelet aggregation [49]. In addition, the C-terminal gamma-chain fibrinogen peptide (HHLGGAKQAGDV) demonstrated the ability to inhibit platelet aggregation and fibrinogen binding to rabbit platelets, through its direct interaction with GPIIb-IIIa [30]. Thus, it is possible that the cleavage of the fibrinogen gamma chain by HF3, detected upon incubation with plasma or isolated fibrinogen, results in the release of the C-terminal peptide, potentially affecting platelet aggregation.

In the same work by Paes Leme et al., 2012, alpha-1-antitrypsin, a serine proteinase inhibitor, was found in lower concentration in the skin injected with HF3 in comparison with the control skin. In the present study, we found that HF3 was able to cleave this protein in vitro, in agreement with its lower abundance in the skin of mice treated with HF3 [19]. Alpha-1-antitrypsin has anti-apoptotic effects and can act as a negative inflammatory regulator [50], thus, considering that HF3 and other metalloproteinases from venoms have pro-inflammatory activity, the degradation of alpha-1-antitrypsin could facilitate the development of inflammation. Peptides derived from alpha-2-macroglobulin and transthyretin were detected in the peptide fraction of plasma treated with HF3, indicating their proteolysis. These proteins were, however, found in higher abundance in the skin of mice injected with HF3, according to Paes Leme et al., 2012 [19], probably due to the increase of blood in the hemorrhagic skin. However, in agreement with the present study, we showed that alpha-2-macroglobulin was not able to inactivate the proteolytic activity of HF3 in vitro, and instead, was partially hydrolyzed by HF3 [16].

Plasminogen, a component of the fibrinolytic system, was identified as a substrate for HF3, but its limited cleavage was only detected in P(LAP-E), with a low number of identified peptides. Plasminogen is a single-chain glycoprotein that circulates in plasma in zymogen form and, when activated by urokinase (u-PA) or tissue plasminogen activator (t-PA) by cleavage of the Arg580-Val581 bond, it is converted to plasmin, and thus is able to cleave fibrin [51]. Angiostatin is an antiangiogenic protein that has been identified as the primary factor controlling the dormant state of cells in a secondary metastatic tumor, inhibiting angiogenesis and resulting in decreased blood flow and reduction in tumor size [52]. Angiostatin is a 38 kDa internal fragment of plasminogen [53], and angiostatin-like molecules can be generated in a variety of ways, including processing of plasminogen by various matrix metalloproteinases [54]. Ho et al., 2002, showed that metalloproteinases from *Bothrops* venoms, when incubated with plasminogen, were capable of generating a product of 38 kDa, and whose N-terminal sequencing evidenced the cleavage in the Ser460-Val461 bond, indicating the generation of an angiostatin-like protein [55]. Here, peptides identified in the peptide fraction of plasma treated with HF3 indicated cleavages in plasminogen close to the regions of initiation and termination of angiostatin. Furthermore, in a recent study, the incubation of HF3 with the isolated plasminogen did generate a product with a molecular mass close to 38 kDa [15], indicating that the limited proteolysis of plasminogen by HF3 indeed generates a protein similar to angiostatin. As angiostatin modulates the rate of plasminogen activation through non-competitive inhibition of the tissue-type plasminogen activator, the cleavage of plasminogen by HF3 and the generation of angiostatin would result in the decrease of the concentration of plasminogen in plasma, and as a consequence, its activity in the fibrinolytic system would be compromised. The impairment in the generation of plasmin, through the decrease of the concentration of plasminogen, would significantly affect the negative feedback of the coagulation cascade and, in turn, would also contribute to the fibrinogen consumption during the coagulopathies triggered by snake envenomation.

Prothrombin (coagulation factor II) was found in higher abundance in the plasma of mice injected with HF3 in the dorsal skin, in comparison to the plasma of mice treated with the control solution [19], by the analysis of plasma proteins subjected to in solution digestion with trypsin, and spectra count by LC-MS/MS. In the present study, prothrombin was identified as cleaved by HF3 by analyzing the plasma peptide fraction. The cleavage of prothrombin has also been shown by incubating the isolated protein with HF3, followed by SDS-PAGE, which revealed products of ~ 28, 30, 35, and 50 kDa [15]. The presence of many peptides from prothrombin in the peptide fraction of plasma treated with HF3 indicates that this protein is rather degraded and not activated by the proteinase, and as a consequence, prothrombin would be unavailable to participate in the blood coagulation process, where it is activated by factor Xa and converted into thrombin. The increase in prothrombin in the plasma of mice injected with HF3, verified in the study by Paes Leme et al., 2012 [19], could be explained as an attempt by the organism (mouse) to counteract, not only the hemorrhagic process, but also the cleavage of the protein itself by HF3, providing more protein to the coagulation cascade.

Alpha-2-antiplasmin is a serine proteinase inhibitor (serpin) that acts to protect fibrin clots from plasmin-mediated cleavage [56]. The plasmin inhibition by the serpin occurs primarily by the binding of the Gln41 residue from its N-terminal region to the Lys342 residue of the fibrin alpha chain, mediated by factor XIIIa. Then, the C-terminal domain of alpha-2-antiplasmin interacts with plasmin, so that its Arg403 aligns and forms a covalent bond with the Ser residue present in the active site of plasmin, forming an inactive enzyme-inhibitor complex [57]. Alpha-2-antiplasmin can be found in plasma either in its mature form (452 amino acids) or in its propeptide-containing form (464 amino acids) [58]. Pro-alpha-2-antiplasmin has inhibitory activity on plasmin, however, its ability to cross-link fibrin is reduced by approximately one third compared to the mature protein [59]. The peptide products generated from alpha-2-antiplasmin by HF3 indicated that both the mature protein and pro-protein forms were cleaved. However, no peptide corresponding to the exact sequence of the pro-domain was identified (M-E-P-L-G-R-Q-L-T-S-G-P), indicating that HF3 would not be able to activate the pro-protein. Furthermore, we detected that HF3 was able to cleave peptides from the N-terminal region of alpha-2-antiplasmin, containing the Gln41 residue involved in the crosslinking to fibrin. Moreover, the C-terminal region was also degraded by the HF3, but the sequence containing the Arg403 residue did not undergo hydrolysis. In view of these findings, we can suggest that HF3 would prevent the binding of alpha-2-antiplasmin to fibrin, however, the effect of HF3 on its inhibitory activity needs further investigation.

In addition to prothrombin, coagulation factor XIII was identified as a substrate of HF3. However, only two peptides (^29^TVELQGVVPR^38^ and ^464^LIVTKQIGGDGMMDITDT^481^) were identified as generated by HF3 from factor XIII (chain A) in P(20-MAP-D), indicating limited proteolysis. The analysis of the activity of HF3 upon isolated factor XIII by SDS-PAGE revealed only a weak protein band of ~28 kDa, which could correspond to the region corresponding to residues 482−732 of the A chain of Factor XIII [15]. Factor XIII is a transglutaminase that circulates in the human plasma as a heterotetramer of two A and two B chains [60]. Activated Factor XIII (FXIIIa) is responsible for stabilizing fibrin by introducing covalent cross-links [61]. FXIIIa is also responsible for cross-linking alpha-2-antiplasmin and fibrin. The activation of FXIII is catalyzed by thrombin that cleaves the Arg38-Gly39 bonds of the A chain of the A2B2 tetramer, leading to chain dissociation and exposure of the cysteine residue of the catalytic site [62]. The identification of the peptide ^29^TVELQGVVPR^38^, which contains the thrombin cleavage site, is an indication of a possible activation of Factor XIII by HF3, however, as other FXIII A chain peptides were identified, it is not possible to state whether the effect of HF3 on this protein is degradation or activation.

Kininogen was identified as a substrate for HF3 in all plasma samples used in this study, and by both bioinformatics methods for protein identification. Peptides derived from both high molecular weight and low molecular weight kininogens have been identified and their extensive proteolysis has been indicated. High molecular weight kininogen participates in the coagulation cascade and the kallikrein-kinin system, whereas low molecular weight kininogen participates only in the latter [63]. These proteins are identical throughout their heavy chains, in the region containing bradykinin, and in the twelve amino acids in the N-terminal region of their light chains. Their heavy chains confer them the function of cysteine proteinase inhibitors [64]. The peptides bradykinin or lysyl-bradykinin are released from both kininogen forms when cleaved by kallikreins. In addition to its vasodilatory function, bradykinin (R-P-P-G-F-S-P-F-R) is an inflammation-mediating peptide [65]. The clotting activity of high molecular weight kininogen resides in its light chain [66], the region through which it binds to prekallikrein and factor XI. Furthermore, the same light chain contains a region rich in histidine, responsible for its binding to negatively charged surfaces [67]. The peptides identified as hydrolysis products generated by HF3 are located in the heavy chain of both kininogens, which would compromise their cysteine proteinase inhibitor function. In addition, the light chain of the high molecular weight kininogen was also cleaved by HF3, which would compromise its role in the coagulation cascade. Finally, we found that the region containing the peptide corresponding to bradykinin is cleaved by HF3, but no peptide corresponding to the exact bradykinin sequence was identified, so further investigations are needed to evaluate whether proteolysis of this region would have any effect on the kallikrein-kinin system.

Ceruloplasmin was also identified as a substrate for HF3, although a low number of peptides was detected as a hydrolysis product, and only in the peptide fraction of P(Alb-D) after treatment with HF3. This protein has a high degree of homology with the factor V (A chain) of the coagulation cascade [68]. Ceruloplasmin carries more than 95% of the copper present in plasma, and its function is related to the regulation of copper and iron homeostasis, in addition to its role in angiogenesis and antioxidant activity [69]. Ceruloplasmin is also an acute phase protein and its plasma level increases in response to inflammation [70,71]. Walker and Fay (1990) reported its ability to bind to protein C, thus protecting factors Va and VIII from inactivation catalyzed by activated protein C. Thus, its hydrolysis by HF3 could be involved in the hemorrhagic process [72].

The complement system, which is part of the innate immune system, is composed of more than 30 plasma proteins and cell surface receptors that react with each other in a range of functions, including direct cell lysis and enhancement of B and T cell responses, and induce a series of inflammatory responses that contribute to fighting infection [73,74,75]. Some proteins are proteinases that are activated in a proteolytic cleavage cascade, similar to the blood coagulation cascade [76]. The activation of the complement system by snake venom components and its role in the envenomation has been shown with venoms from the *Elapidae* and *Viperidae* families [77,78]. Among other potential new targets for HF3, this study identified some components of the complement cascade: C1r; C1s; C3; C4 and C9; C4b binding protein (alpha chain); complement factor H; and factor H-related protein 1. Cleavage of components C3 and C4 was confirmed by incubation of HF3 with these isolated proteins [15], indicating that they would not be available in plasma to participate in the complement cascade activation process, hence HF3 would play a role as a modulator of this cascade of proteinases.

Peptides from clusterin (apoliprotein J) were identified in the peptide fraction of P(W), P(Alb-D), P(20-MAP-D), and P(LAP-E) after incubation with HF3, indicating an extensive degradation of this protein. Clusterin is a chaperone protein with anti-inflammatory and cytoprotective activity, which inhibits MMP-9, MMP-2, MMP-3, and MMP-7 [79], so its proteolysis by HF3 could prevent its anti-inflammatory activity. Further, it is possible to suggest that, opposite to what happens with MMPs, clusterin would not be an inhibitor of HF3.

The results of the present study also showed potential new HF3 targets, such as alpha-2-HS-glycoprotein, or fetuin-A, which was shown as extensively degraded by HF3. This protein functions as an important component of several physiological or pathological mechanisms, including vascular calcification, bone metabolism, insulin resistance, keratinocyte migration, and breast cancer tumor cell proliferative signaling [80]. In response to acute inflammation, alpha-2-HS-glycoprotein appears to act as an anti-inflammatory modulator. Alpha-2-HS-glycoprotein has been shown to suppress the excessive release of tumor necrosis factor from activated macrophages [80,81,82]. Hence, the proteolysis of alpha-2-HS-glycoprotein by HF3 would impair its anti-inflammatory activity. Meprins are zinc-dependent, astacin-like metalloproteinases that play a pivotal role in inflammation by activating cytokines, and the potential role of endogenous meprin inhibitor has also been attributed to alpha-2-HS-glycoprotein [80,83,84]. Guerranti et al., 2010 [38], reported the proteolysis of alpha-2-HS-glycoprotein after incubation of human plasma with *Echis carinatus* venom, however, the component of the venom responsible for its degradation has not been investigated. To our knowledge, there are no reports on the cleavage of alpha-2-HS-glycoprotein by other venom metalloproteinases, and its degradation by HF3 would potentially have a pro-inflammatory effect.

In the study by Paes Leme et al., 2012 [19], it was found that apolipoprotein A-II, the second major component of HDL particles, was clearly degraded in the hemorrhagic process generated by HF3. Here we show that not only apolipoprotein A-II, but also other proteins of the same family (apolipoprotein AI, A-IV, CI, C-II, C-III, E, F, and L1), were detected as substrates of HF3. Apolipoproteins bind and transport lipids, and members of classes A, C, and E play an important role in lipoprotein metabolism and in inflammatory processes [85]. Previous studies have shown that apolipoproteins AI and E are cleaved by matrix metalloproteinase 14 (MMP-14) [39], whereas apolipoproteins C-II and A-IV are cleaved by matrix metalloproteinases 7 and 14 [86,87,88]. The cleavage of apolipoproteins A-I and A-II by metalloproteinases from the venom of *Cerastes cerastes* was observed by El-Asmar and Swaney (1988) [89], and the cleavage of apolipoprotein A-I by proteinases in the venom of *Echis carinatus* was also reported by Guerranti et al., 2010 [38]. However, the role of proteolysis of these apolipoproteins in the context of envenomation is not well established, and a hypothesis that can be suggested is that the degradation products of these proteins could play a role in modulating the pro-inflammatory activity of venom metalloproteinases. Whether this modulation would be characterized by the increase or by the limitation of the pro-inflammatory effects is a matter of future investigation.

Gelsolin was identified as a substrate for HF3 in P(W), P(20-MAP-D), and P(LAP-E), and was extensively degraded. In another study by our group on the degradation of proteins secreted by fibroblasts, the cleavage of gelsolin by HF3 was also identified by the TAILS approach [20]. Plasma gelsolin, together with the vitamin D-binding protein, forms part of the actin scavenger system [90,91]. Excessive release of intracellular actin or decreased activity of the scavenger system is associated with pathological conditions such as small blood vessel obstruction, clots, endothelial damage, hepatic necrosis, and septic shock [92]. Studies using degradomics approaches showed that gelsolin is a substrate of matrix metalloproteinases, and MMP-3 is able to cleave it more efficiently, followed by MMP-2, MMP-1, and MMP-14 [93]. Thus, as in the case of MMPs, gelsolin cleavage by HF3 could affect the actin scavenging system and promote pathological conditions induced by an excess of unremoved extracellular actin.

Many peptides from the H1, H2, H3, and H4 heavy chains of inter-alpha trypsin inhibitor family proteins were identified in the peptide fraction of plasma after incubation with HF3, indicating extensive cleavage of these polypeptide chains. Proteins of the inter-alpha trypsin inhibitor family are composed of a common light chain (the chondroitin sulfate proteoglycan bikunin), and various heavy chains [94,95]. Bikunin shows inhibitory activity on a wide spectrum of proteinases, including some of pathological importance, such as trypsin, chymotrypsin, plasmin, elastase, and cathepsin. Bikunin is also recognized as an anti-inflammatory mediator [96]. The binding of the heavy chains of the inter-alpha trypsin inhibitor to hyaluronan may be involved in the stabilization and integrity of the extracellular matrix, and provides anti-inflammatory properties [97,98,99]. Catanese and Kress (1985) reported on the in vitro cleavage of the inter-alpha trypsin inhibitor by metalloproteinases from viperid, colubrid, and elapid venoms, generating products still capable of inhibiting trypsin [100]. The degradation of H1−H4 chains of inter-alpha trypsin inhibitor by HF3 would likely not impair the ability of its light chain to inhibit serine proteinases, but would affect its anti-inflammatory activity.

The histidine-rich glycoprotein was also identified as cleaved by HF3, however, this cleavage was very specific and generated peptides within the sequence HSHGPPLPQGPPPLLPM of its proline-rich region [101]. Histidine-rich glycoprotein is found in plasma, leukocytes, platelet α-granules, and megakaryocytes. It contains various binding domains, and interacts with the heme group, plasminogen, heparin, fibrinogen, thrombospondin, immunoglobulin G, complement components, and Zn^2+^ ions [102]. Histidine-rich glycoprotein can also interact with cell-associated molecules, including Fcγ receptors, heparan sulfate, and phospholipids [102]. In the context of this study, the most interesting aspect of the histidine-rich glycoprotein lies in its ability to modulate components of the coagulation cascade. It can bind to heparin released by mast cells, preventing it from inhibiting the procoagulant activity of monocytes at the site of inflammation and thrombosis [101,103]. Some studies have demonstrated the profibrinolytic effect of histidine-rich glycoprotein, by its ability to bind to plasminogen, stimulating its cleavage by plasmin [104]. Other studies, on the other hand, have investigated its action as an antifibrinolytic agent, as the histidine-rich glycoprotein binding to plasminogen could interfere with the interaction of plasminogen with fibrin clots, thus inhibiting plasmin-mediated fibrinolysis [101,105]. Thus, the cleavage of histidine-rich glycoprotein by HF3 would have a variety of implications in the context of hemorrhage, mostly related to its role in regulating hemostasis.

Other proteins were detected as cleaved by HF3, but a low number of peptides were found as hydrolysis products, and mostly in only one type of plasma preparation: carboxypeptidase B2; collectin-11; ficolin-2; galectin-3 binding protein; glutathione peroxidase 3; histone H2B type FS; hyaluronan binding protein; MAX gene-associated protein isoform 3; pigmented epithelial-derived factor; pregnancy zone protein; proline-rich acidic proteins; amyloid A protein -4 serum; immunoglobulins; tetranectin; and serum paraoxonase/arylesterase. These proteins do not have a defined role in the generation of hemorrhage, and thus the implications of their hydrolysis by HF3 need further investigation.

## 4. Conclusions

The human plasma depletion methods used in this study provided heterogeneous samples with respect to the range of dynamic protein concentration, which were compatible with the peptidomic analysis, and generated complementary results for the elucidation of the HF3 degradome. The two bioinformatics approaches used for the analysis of the peptide fraction gave robustness to the set of obtained results, since most proteins identified as HF3 substrates were detected by both. The determination of the primary specificity of HF3 on protein substrates showed that Leu at P1′ is a major determinant of HF3 primary specificity, which agrees with previous studies using peptides, and reinforces the importance of this residue at P1′, regardless of the substrate structure.

**Figure 9:**
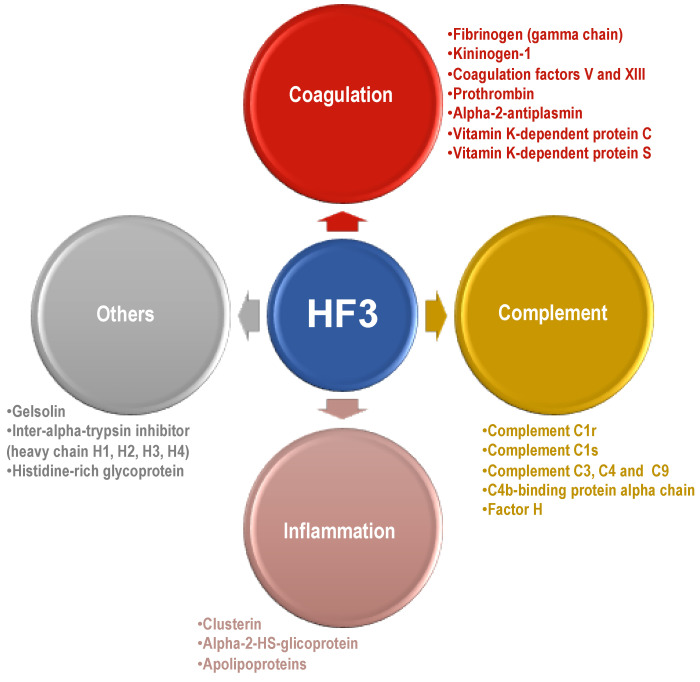
Schematic representation of some of the new substrates of HF3 revealed in this study, according to biological function.

As a result of this study, knowledge about the HF3 substrate repertoire in human plasma has been expanded in terms of number, as well as protein classes and functions (Figure 9). Taken together, the results illustrate the proteolytic signature of human plasma in the context of HF3-induced hemorrhage. By acting on distinct substrates, which are part of a highly connected biological circuit, the proteolytic signaling triggered by HF3 may not be fully anticipated by the results of in vitro incubation with single substrates. Actually, the activated/impaired biological pathways involved in the hemorrhagic and pro-inflammatory effects of SVMPs are the result of complex signaling circuits, which are significantly affected by limited proteolysis and protein degradation. In this regard, it was interesting to note that the hydrolysis of some proteins by HF3 seems to lead to antagonistic results, such as the hydrolysis of fibrinogen and plasminogen, which play roles in different steps of blood coagulation and fibrinolysis. In general, the characterization of HF3 substrate degradome in the human plasma suggests that it acts in a dysregulated manner, refractory to plasma inhibitors, causing an imbalance in hemostasis.

The hydrolysis of human plasma proteins by an uncontrolled, exogeneous metalloproteinase has a direct impact in the plasma proteome, and it can be hypothesized that some hydrolysis products could also play synergistic roles in the pro-inflammatory and hemorrhagic processes generated by HF3.

## 5. Materials and Methods

### 5.1. HF3

HF3 (Uniprot entry Q98UF9) was purified as described previously (Oliveira et al., 2009) from *B. jararaca* venom provided by the Laboratory of Herpetology of Butantan Institute (São Paulo, Brazil), and identified by trypsin digestion and mass spectrometric (LC–MS/MS) analysis.

### 5.2. Analytical Procedures

Protein and peptide contents were quantified using, respectively, Bradford assay (Sigma-Aldrich, St. Louis, MO, USA) and micro-BCA assay (Pierce) kits according to the manufacturers’ recommendations. SDS–PAGE was carried out according to Laemmli (1970) [106]. Silver staining was carried out according to Mortz et al., 2001 [107].

### 5.3. Human Plasma

Procedures using human blood in this study were approved by ethics committee of Instituto Federal de Educação, Ciência e Tecnologia de São Paulo, Brazil, and registered under CAAE 19892213.7.0000.5473. Human blood was collected from three volunteers who declared they had not used painkillers for at least 10 days. In order to obtain human plasma, blood was collected and anticoagulated with 0.1 vol. of 3.8% sodium citrate and centrifuged at 1000× *g* for 10 min at 4 °C. Whole plasma samples (n = 3) were designated as P(W).

### 5.4. Albumin-Depleted Human Plasma

To deplete serum albumin from human plasma, we employed Blue Sepharose CL-6B resin affinity chromatography (Sigma-Aldrich, St. Louis, MO, USA; [108,109]). Three independent experiments were carried out with plasma samples from three individuals. The procedure was performed using a microtube with a filter, according to the manufacturer’s instructions. In the upper part of the filter microtube, 0.5 mL of resin was added, and steps for resin conditioning, binding and elution, and resin reconstitution were carried out by fractioning the total volume required in 0.5 mL aliquots, added to the top of the microtube, followed by mild manual agitation and centrifugation for 1 min at 150× *g*, with disposal or storage of the eluted solution conducted according to the stage. The following solutions were added: (i) conditioning: 2.5 mL of ultrapure water and 4.0 mL of binding buffer (20 mM HEPES, pH 7.4), with disposal of the eluted solution; (ii) binding: the lower outlet duct of the micro tube containing the resin was closed, and 40 μL of plasma diluted in 310 μL of binding buffer was loaded. The tube was kept at room temperature for 30 min under agitation. After centrifugation, the eluted material, corresponding to the plasma depleted of albumin, was stored. For the elution of proteins bound to the resin (eluate), 4 mL of 2 M NaCl were added to the resin, and the eluted solution was stored. For resin reconstitution, 1 mL of 6 M guanidine hydrochloride and 4 mL of binding buffer were applied to the resin, and the eluted solution was discarded. Albumin-depleted plasma samples were designated as P(Alb-D).

### 5.5. Human Plasma Depleted of 20 Most Abundant Proteins

Depletion of the 20 most abundant proteins from human plasma was performed with the ProteoPrep 20 Plasma Immunodepletion Kit (Sigma-Aldrich, St. Louis, MO, USA), according to the manufacturer’s instructions. Three independent experiments were carried out with plasma samples from three individuals. Briefly, samples of 8 μL of human plasma were diluted to 100 µL with phosphate-buffered saline (PBS) and loaded to a micro-spin column, previously equilibrated with PBS. After incubation for 20 min at room temperature, the non-bound protein fraction was recovered by centrifugation at 2000 rpm for 1 min and the flow-through, containing plasma depleted of the 20 most abundant proteins P(20-MAP-D), was collected in a clean tube. The remaining unbound proteins were washed twice by adding 100 µL of PBS, centrifuging, and collecting the wash in the same tube. The depletion procedure was repeated five times, and flow-throughs were pooled and then concentrated using a Centricon YM-3 filter (Millipore). After each depletion procedure, the micro-spin column was regenerated with 2 mL of 0.1 M Glycine-HCl, pH 2.5, and TWEEN 20 in order to elute bound proteins, and stored at 5 °C in 5 mL of PBS with the addition of 10 µL of ProteoPrep Preservative Concentrate. Human plasma depleted of 20 most abundant proteins was designated as P(20-MAP-D).

### 5.6. Human Plasma Enriched in Low-Abundance Proteins

The enrichment of low-abundant proteins in human plasma was performed with the ProteoMiner Protein Enrichment Kit (Bio-Rad) [37] according to the manufacturer’s instructions. Three independent experiments were carried out with plasma samples from three individuals. In brief, 200 μL of human plasma was loaded onto the column previously conditioned with PBS, pH 7.4, and incubated for 2 h at room temperature. After centrifugation, the flow-through fraction was stored for further analysis. The fraction that bound to the resin, containing the low-abundant proteins, was eluted with 8 M urea containing 2% CHAPS. For desalinization, the proteins in solution were precipitated by the addition of eight volumes of cold acetone and one volume of cold methanol, and stored for 12 h at −20 °C. After centrifugation for 10 min at 14,000× *g* at 4 °C, the precipitate was washed with cold methanol and resuspended in 60 μL of 100 mM NaOH and 340 μL of 50 mM HEPES, pH 7.5. Plasma enriched of low-abundant proteins was designated as P(LAP-E).

### 5.7. Proteolytic Activity of HF3 on Human Plasma

For each of the four types of human plasma preparations, three biological replicates were performed. For each experiment, P(Alb-D), P(20-MAP-D), and P(LAP-E); 50 μg were separately incubated with 0.5 μg HF3 (1:100; *w/w*) in 250 mM ammonium acetate containing CaCl_2_ 1 mM for 2 h at 37 °C. P(W) (200 μg) was incubated with 2 μg HF3 under the same conditions. Samples of P(Alb-D), P(20-MAP-D), P(LAP-E), and P(W) were incubated without HF3, as a control, under identical conditions. Reactions were stopped by adding eight volumes of cold acetone and one volume of cold methanol, and incubated for 12 h at −20 °C. Peptide fractions (supernatant) were obtained by centrifugation at 14,000 g for 10 min at −4 °C, and subsequently dried using a SpeedVac concentrator. The protein fraction (precipitate) was separated and stored at −20 °C until use.

### 5.8. LC-MS/MS Analysis of the Plasma Peptide Fraction

Previously to LC-MS/MS analysis, plasma peptide fractions containing hydrolysis products resulting from the proteolytic activity of HF3 were subjected to removal of traces of detergent using Macro Spin Columns (Harvard Apparatus). Samples were then desalted with Sep-Pak Light C18 (Waters) cartridges, vacuum dried, and resuspended in 20 μL of 0.1% formic acid. Aliquots of 10 µL were separated by RP-HPLC on an EASY-nLC II (Thermo Scientific, Waltham, MA, USA) using a column (75 μm i.d. × 10 cm) packed with 5 μm C18 beads (Phenomenex), and coupled to an LTQ-Orbitrap Velos mass spectrometer (Thermo Fisher Scientific, Waltham, MA, USA). The gradient was 5–40% acetonitrile in 0.1 M formic acid over 90 min, at a flow rate of 300 nL/min. The mass spectrometer was operated in data dependent mode, in which one full MS scan was acquired in the m/z range of 400–2000 at 60,000 resolution, followed by MS/MS acquisition using high-energy collision dissociation of the six most intense ions from the MS scan, at 15,000 resolution. A dynamic peak exclusion was applied to avoid the same m/z of being selected for the next 25 s, using a ± 1 Da mass tolerance window around the precursor ion mass.

### 5.9. Mass Spectrometry Data Analysis

Two strategies were employed for the identification of peptides originated from HF3 cleavage of plasma proteins. In the first, we used the Mascot program, and validated the results using the PeptideProphet and ProteinProphet tools of the Trans-Proteomic Pipeline (TPP) platform (Mascot/TPP). In the second, de novo sequencing, searching the database, and validating the results were carried out using the Peaks Studio 7 program (Peaks).

Mascot/TPP: Acquired MS/MS raw data were converted to the mgf and mzXML format using MS-Convert. Database searches were performed against the human UniProtKB/Swiss-Prot protein database (available at http://www.uniprot.org; accessed on 3 February 2014) using Mascot 2.4.1 (Matrix Science) with the following parameters: no enzyme specificity indicated; 10 ppm precursor tolerance; 20 mmu fragment ion tolerance; variable Met oxidation (+15.9949 Da); variable *N* terminal acetylation (+42.0105 Da); and variable Asn and Gln deamidation (+0.9840 Da). Mascot search results were further processed using the Trans-Proteomic Pipeline (TPP, version 4.6) (Keller and Shteynberg, 2011). Peptides were included in the analysis if they were identified at a false discovery rate (FDR) of ≤1% at peptide level (PeptideProphet), and (ii) proteins at an FDR of ≤1% at protein level (ProteinProphet).

Peaks: Acquired MS/MS raw data were imported into Peaks Studio 7 software (Ma et al., 2003). De novo analysis was performed with the following parameters: no enzyme specificity indicated; 10 ppm precursor tolerance; 0.02 Da fragment ion tolerance; variable Met oxidation (+15.9949 Da); variable *N* terminal acetylation (+42.0105 Da); and variable Asn and Gln deamidation (+0.9840 Da). After de novo sequencing, a database search (Peaks DB) was performed against the human UniProtKB/Swiss-Prot protein database (available at http://www.uniprot.org; accessed on 10 February 2014), using the same parameters. Peptides were included in the analysis if they were identified at a Peaks DB FDR of ≤1%.

### 5.10. Identification of Peptides Generated the Incubation of Fibrinogen with HF3

Fibrinogen (50 μg) was incubated with HF3 (0.5 μg) in 0.025 M Tris-HCl, pH 7.5, 5 mM CaCl_2_ for 2 h at 37 °C. The reaction was stopped by adding eight volumes of cold acetone and one volume of cold methanol, and stored for 12 h at −20 °C. After centrifugation at 14,000× *g* for 10 min at 4 °C, the supernatant, corresponding to the peptide fraction, was dried in a SpeedVac concentrator, subjected to desalination procedures using Sep-Pak Light C18 cartridges, vacuum dried, and resuspended in 20 μL of 0.1% formic acid for analysis by LC-MS/MS, as described above, and a database search using the Peaks Studio 7 program.

### 5.11. Validation of HF3 Substrates in Human Plasma by Incubation with HF3

Apolipoprotein A-IV, clusterin, α-2-antiplasmin, kininogen, and transthyretin (2 μg; Sigma-Aldrich, St. Louis, MO, USA) were incubated with HF3 (200 ng) (1:10 (*w/w*) enzyme-to-substrate ratio) in 0.05 M Tris-HCl, pH 8.0, 1.0 mM CaCl_2_ for 2 h at 37 °C. Apoliprotein E (4 μg; Sigma-Aldrich, St. Louis, MO, USA) was incubated with HF3 (400 ng) (1:10 (*w/w*) enzyme-to-substrate ratio) in the same buffer. A sample of each protein was incubated without enzymes under identical conditions. Reactions were stopped by adding a Laemmli sample buffer, and subjected to SDS-PAGE.

## Figures and Tables

**Table 1:** HF3 substrates revealed by the analysis of P(W) peptide fraction by LC-MS/MS. Only proteins that showed peptides identified exclusively in the sample of plasma treated with HF3 were considered. The boldface indicates the ten most degraded proteins according to the number of identified spectra.

		Number of Spectra * (exp. 1/exp. 2/exp. 3)	
Identification	Protein	Mascot/TPP	PEAKS
ACTB_HUMAN	Actin cytoplasmic 1	0/0/0	7/3/0
A1AT_HUMAN	Alpha-1-antitrypsin	1/0/1	6/10/5
A2AP_HUMAN	Alpha-2-antiplasmin	12/13/13	20/24/18
**FETUA_HUMAN**	**Alpha-2-HS-glycoprotein**	**75/94/92**	**270/437/391**
A2MG_HUMAN	Alpha-2-macroglobulin	2/0/2	9/4/8
APOA1_HUMAN	Apolipoprotein A-I	9/25/14	43/58/50
**APOA2_HUMAN**	**Apolipoprotein A-II**	**13/30/19**	**63/70/60**
APOA4_HUMAN	Apolipoprotein A-IV	5/6/7	23/21/16
APOC1_HUMAN	Apolipoprotein C-I	0/0/0	4/5/0
APOC2_HUMAN	Apolipoprotein C-II	7/17/11	30/41/30
APOC3_HUMAN	Apolipoprotein C-III	9/19/13	34/43/30
APOE_HUMAN	Apolipoprotein E	3/4/3	10/9/10
APOF_HUMAN	Apolipoprotein F	2/5/3	7/11/12
APOL1_HUMAN	Apolipoprotein L1	4/9/5	36/38/35
**CLUS_HUMAN**	**Clusterin**	**17/42/29**	**89/108/90**
FA5_HUMAN	Coagulation factor V	0/0/0	3/0/3
C1R_HUMAN	Complement C1r subcomponent	1/2/2	18/15/15
C1S_HUMAN	Complement C1s subcomponent	3/3/2	26/20/16
CO3_HUMAN	Complement C3	9/18/16	20/26/21
**CO4A_HUMAN**	**Complement C4-A**	**19/30/24**	**61/76/67**
CO9_HUMAN	Complement component C9	0/2/1	3/4/0
CFAH_HUMAN	Complement factor H	1/2/0	3/2/0
**FIBA_HUMAN**	**Fibrinogen alpha chain**	**126/221/163**	**369/602/462**
**FIBB_HUMAN**	**Fibrinogen beta chain**	**16/52/32**	**39/53/45**
FINC_HUMAN	Fibronectin	7/12/6	29/29/22
GELS_HUMAN	Gelsolin	5/8/7	28/21/25
HRG_HUMAN	Histidine-rich glycoprotein	10/10/8	24/55/39
H2BFS_HUMAN	Histone H2B type F-S	0/0/0	4/1/0
IGHG1_HUMAN	Ig gamma-1 chain C region	0/5/1	6/8/7
IGHM_HUMAN	Ig mu chain C region	2/5/2	6/6/6
ITIH1_HUMAN	Inter-alpha-trypsin inhibitor heavy chain H1	6/5/6	17/17/19
**ITIH2_HUMAN**	**Inter-alpha-trypsin inhibitor heavy chain H2**	**51/92/79**	**204/230/224**
ITIH3_HUMAN	Inter-alpha-trypsin inhibitor heavy chain H3	1/2/1	6/4/5
**ITIH4_HUMAN**	**Inter-alpha-trypsin inhibitor heavy chain H4**	**24/37/28**	**93/103/94**
KNG1_HUMAN	Kininogen-1	8/8/10	33/21/30
PEDF_HUMAN	Pigment epithelium-derived factor	3/4/3	5/5/4
PZP_HUMAN	Pregnancy zone protein	0/1/2	5/5/5
**THRB_HUMAN**	**Prothrombin**	**17/25/20**	**63/62/65**
ALBU_HUMAN	Serum albumin	0/0/0	0/3/2
TTHY_HUMAN	Transthyretin	6/17/17	43/43/43
**VTNC_HUMAN**	**Vitronectin**	**18/31/24**	**74/78/71**

* Only spectra identified exclusively in the HF3-treated samples were considered.

**Table 2:** HF3 substrates revealed by the analysis of P(Alb-D) peptide fraction by LC-MS/MS. Only proteins that showed peptides identified exclusively in the sample of plasma treated with HF3 were considered. The boldface indicates the ten most degraded proteins according to the number of identified spectra.

		Number of Spectra * (exp. 1/exp. 2/exp. 3)	
Identification	Protein	Mascot/TPP	PEAKS
ACTG_HUMAN	Actin cytoplasmic 1	0/0/0	1/0/1
**A1AT_HUMAN**	**Alpha-1-antitrypsin**	**1/10/11**	**16/24/25**
**A2AP_HUMAN**	**Alpha-2-antiplasmin**	**10/12/11**	**17/18/20**
**FETUA_HUMAN**	**Alpha-2-HS-glycoprotein**	**84/109/117**	**219/226/230**
**APOA1_HUMAN**	**Apolipoprotein A-I**	**13/0/1**	**33/8/6**
APOA2_HUMAN	Apolipoprotein A-II	5/3/1	17/13/11
APOA4_HUMAN	Apolipoprotein A-IV	8/5/7	13/13/15
APOC2_HUMAN	Apolipoprotein C-II	4/3/2	11/8/8
**APOC3_HUMAN**	**Apolipoprotein C-III**	**6/12/10**	**21/25/27**
APOE_HUMAN	Apolipoprotein E	3/2/2	2/4/4
APOL1_HUMAN	Apolipoprotein L1	0/4/1	4/8/10
CERU_HUMAN	Ceruloplasmin	0/2/3	2/6/9
**CLUS_HUMAN**	**Clusterin**	**1/2/2**	**5/16/12**
CO3_HUMAN	Complement C3	0/2/4	0/2/8
**FIBA_HUMAN**	**Fibrinogen alpha chain**	**0/3/14**	**2/13/41**
**FIBB_HUMAN**	**Fibrinogen beta chain**	**7/14/20**	**13/20/28**
FINC_HUMAN	Fibronectin	0/1/2	0/0/0
GPX3_HUMAN	Glutathione peroxidase 3	0/0/0	5/7/0
IGHG1_HUMAN	Ig gamma-1 chain C region	2/1/0	0/0/0
**ITIH2_HUMAN**	**Inter-alpha-trypsin inhibitor heavy chain H2**	**2/7/18**	**7/26/42**
ITIH4_HUMAN	Inter-alpha-trypsin inhibitor heavy chain H4	0/4/4	0/8/7
MGAP_HUMAN	Isoform 3 of MAX gene-associated protein	4/2/0	0/0/0
KNG1_HUMAN	Kininogen-1	1/2/1	2/8/6
THRB_HUMAN	Prothrombin	1/2/5	2/6/12
**PON1_HUMAN**	**Serum paraoxonase/arylesterase 1**	**7/15/21**	**27/36/52**
**TTHY_HUMAN**	**Transthyretin**	**27/33/34**	**44/57/68**

* Only spectra identified exclusively in the HF3-treated samples were considered.

**Table 3:** HF3 substrates revealed by the analysis of P(20-MAP-D) peptide fraction by LC-MS/MS. Only proteins that showed peptides identified exclusively in the sample of plasma treated with HF3 were considered. The boldface indicates the ten most degraded proteins according to the number of identified spectra.

		Number of Spectra * (exp. 1/exp. 2/exp. 3)	
Identification	Protein	Mascot/TPP	PEAKS
ACTB_HUMAN	Actin cytoplasmic 1	6/0/2	18/0/2
**A2AP_HUMAN**	**Alpha-2-antiplasmin**	**70/28/20**	**81/39/30**
**FETUA_HUMAN**	**Alpha-2-HS-glycoprotein**	**270/150/134**	**454/232/245**
A2MG_HUMAN	Alpha-2-macroglobulin	5/4/3	7/6/4
**APOA1_HUMAN**	**Apolipoprotein A-I**	**69/39/36**	**91/58/62**
**APOA2_HUMAN**	**Apolipoprotein A-II**	**39/19/21**	**56/30/37**
**APOA4_HUMAN**	**Apolipoprotein A-IV**	**28/48/15**	**56/71/31**
APOC1_HUMAN	Apolipoprotein C-I	0/0/0	2/2/0
APOC2_HUMAN	Apolipoprotein C-II	39/4/10	46/6/18
APOC3_HUMAN	Apolipoprotein C-III	55/9/10	68/14/17
APOE_HUMAN	Apolipoprotein E	4/9/2	8/11/7
APOF_HUMAN	Apolipoprotein F	17/7/2	26/4/0
APOL1_HUMAN	Apolipoprotein L1	8/4/7	8/3/19
CBPB2_HUMAN	Carboxypeptidase B2	0/0/0	1/0/4
CLUS_HUMAN	Clusterin	5/2/8	10/4/30
F13A_HUMAN	Coagulation factor XIII A chain	5/0/1	5/0/1
C1R_HUMAN	Complement C1r subcomponent	5/0/2	0/0/0
CO3_HUMAN	Complement C3	5/2/3	5/2/6
CO4A_HUMAN	Complement C4-A	6/0/10	9/0/15
ECM1_HUMAN	Extracellular matrix protein 1	0/0/0	4/3/0
**FIBA_HUMAN**	**Fibrinogen alpha chain**	**159/50/83**	**162/0/122**
**FIBB_HUMAN**	**Fibrinogen beta chain**	**31/38/14**	**34/44/17**
FINC_HUMAN	Fibronectin	4/1/8	6/5/22
**GELS_HUMAN**	**Gelsolin**	**49/18/14**	**68/23/42**
HRG_HUMAN	Histidine-rich glycoprotein	2/6/14	5/7/29
IGHG1_HUMAN	Ig gamma-1 chain C region	4/0/9	4/0/12
**ITIH2_HUMAN**	**Inter-alpha-trypsin inhibitor heavy chain H2**	**20/18/34**	**31/23/69**
ITIH3_HUMAN	Inter-alpha-trypsin inhibitor heavy chain H3	0/0/0	2/0/9
**ITIH4_HUMAN**	**Inter-alpha-trypsin inhibitor heavy chain H4**	**131/44/38**	**174/56/80**
FIBG_HUMAN	Isoform Gamma-A of Fibrinogen gamma chain	2/19/0	3/19/0
KNG1_HUMAN	Kininogen-1	7/1/3	17/3/9
THRB_HUMAN	Prothrombin	1/2/23	1/2/61
SAA4_HUMAN	Serum amyloid A-4 protein	0/0/0	7/1/0
TETN_HUMAN	Tetranectin	5/2/5	5/2/6
TTHY_HUMAN	Transthyretin	9/2/10	10/2/15
VTNC_HUMAN	Vitronectin	33/9/11	43/12/15

* Only spectra identified exclusively in the HF3-treated samples were considered.

**Table 4:** HF3 substrates revealed by the analysis of P(LAP-E) peptide fraction by LC-MS/MS. Only proteins that showed peptides identified exclusively in the sample of plasma treated with HF3 were considered. The boldface indicates the ten most degraded proteins according to the number of identified spectra.

		Number of Spectra * (exp 1/exp 2/exp 3)	
Identification	Protein	Mascot/TPP	PEAKS
A1AT_HUMAN	Alpha-1-antitrypsin	3/15/1	5/18/3
A2AP_HUMAN	Alpha-2-antiplasmin	1/2/0	2/4/0
**FETUA_HUMAN**	**Alpha-2-HS-glycoprotein**	**32/31/30**	**51/60/49**
**APOA1_HUMAN**	**Apolipoprotein A-I**	**80/127/17**	**171/258/37**
**APOA2_HUMAN**	**Apolipoprotein A-II**	**37/69/20**	**56/132/26**
**APOA4_HUMAN**	**Apolipoprotein A-IV**	**52/86/11**	**124/201/20**
APOC1_HUMAN	Apolipoprotein C-I	11/17/0	34/45/2
APOC2_HUMAN	Apolipoprotein C-II	22/21/5	27/32/9
**APOC3_HUMAN**	**Apolipoprotein C-III**	**19/51/5**	**43/88/11**
**APOE_HUMAN**	**Apolipoprotein E**	**29/9/4**	**57/30/13**
APOF_HUMAN	Apolipoprotein F	1/2/0	1/2/0
APOL1_HUMAN	Apolipoprotein L1	0/0/0	0/3/2
C4BPA_HUMAN	C4b-binding protein alpha chain	0/0/0	3/5/0
CBPB2_HUMAN	Carboxypeptidase B2	1/0/1	1/0/1
CLUS_HUMAN	Clusterin	1/18/0	7/49/0
CO1A2_HUMAN	Collagen alpha-2(I) chain	0/0/0	1/0/1
COL11_HUMAN	Collectin-11	0/2/1	3/5/2
C1QB_HUMAN	Complement C1q subcomponent subunit B	0/0/0	1/0/1
C1S_HUMAN	Complement C1s subcomponent	0/0/0	1/3/0
CO3_HUMAN	Complement C3	8/9/7	13/22/21
CO4A_HUMAN	Complement C4-A	4/14/4	17/30/11
CFAH_HUMAN	Complement factor H	0/3/2	0/7/2
FHR1_HUMAN	Complement factor H-related protein 1	0/0/0	3/8/0
**FIBA_HUMAN**	**Fibrinogen alpha chain**	**82/141/40**	**149/241/63**
**FIBB_HUMAN**	**Fibrinogen beta chain**	**160/29/9**	**84/50/7**
FINC_HUMAN	Fibronectin	4/7/6	4/10/15
FCN2_HUMAN	Ficolin-2	1/2/0	2/4/0
LG3BP_HUMAN	Galectin-3-binding protein	0/0/0	2/2/0
GELS_HUMAN	Gelsolin	3/2/1	5/6/2
GPX3_HUMAN	Glutathione peroxidase 3	1/0/1	1/2/1
HRG_HUMAN	Histidine-rich glycoprotein	0/0/0	1/5/0
HABP2_HUMAN	Hyaluronan-binding protein 2	2/3/0	2/6/0
IGHG1_HUMAN	Ig gamma-1 chain C region	0/2/4	0/6/10
IGKC_HUMAN	Ig kappa chain C region	1/9/0	3/14/1
KV309_HUMAN	Ig kappa chain V-III region VG (Fragment)	0/0/0	1/6/0
KV402_HUMAN	Ig kappa chain V-IV region Len	0/0/0	1/2/0
LV101_HUMAN	Ig lambda chain V-I region V	0/0/0	1/3/2
LV403_HUMAN	Ig lambda chain V-IV region Hil	0/0/0	0/3/1
LAC2_HUMAN	Ig lambda-2 chain C regions	3/4/0	8/12/9
IGLL5_HUMAN	Immunoglobulin lambda-like polypeptide 5	0/0/0	0/8/12
ITIH1_HUMAN	Inter-alpha-trypsin inhibitor heavy chain H1	2/4/0	3/8/0
**ITIH2_HUMAN**	**Inter-alpha-trypsin inhibitor heavy chain H2**	**23/24/2**	**35/42/3**
ITIH4_HUMAN	Inter-alpha-trypsin inhibitor heavy chain H4	10/7/2	21/20/8
FIBG_HUMAN	Isoform Gamma-A of fibrinogen gamma chain	8/9/0	19/25/0
KNG1_HUMAN	Kininogen-1	2/3/0	6/6/3
PLMN_HUMAN	Plasminogen	0/0/0	1/6/0
PRAP1_HUMAN	Proline-rich acidic protein 1	0/0/0	4/1/2
THRB_HUMAN	Prothrombin	7/20/5	21/43/12
ALBU_HUMAN	Serum albumin	1/10/1	2/25/6
SAA4_HUMAN	Serum amyloid A-4 protein	4/3/0	14/11/1
TTHY_HUMAN	Transthyretin	2/5/0	7/13/3
PROC_HUMAN	Vitamin K-dependent protein C	0/0/0	0/1/1
PROS_HUMAN	Vitamin K-dependent protein S	2/1/0	4/5/1
**VTNC_HUMAN**	**Vitronectin**	**19/43/13**	**41/85/20**

* Only spectra identified exclusively in the HF3-treated samples were considered.

**Table 5:** 61 proteins identified as cleaved by HF3 in human plasma.

Actin Cytoplasmic-1	Fibrinogen
Alpha-1-antitrypsin	Fibronectin
Alpha-2-antiplasmin	Ficolin-2
Alpha-2-HS-glycoprotein	Galectin-3-binding protein
Apha-2-macroglobulin	Gelsolin
Apolipoprotein A-I	Glutathione peroxidase 3
Apolipoprotein A-II	Histidine-rich glycoprotein
Apolipoprotein A-IV	Histone H2B type F-S
Apolipoprotein C-I	Hyaluronan-binding protein 2
Apolipoprotein C-II	Ig gamma
Apolipoprotein C-IIII	Ig kappa
Apolipoprotein E	Ig lambda
Apolipoprotein F	Ig mu chain C region
Apolipoprotein L1	Ig lambda-like polypeptide 5
C4b-binding protein alpha chain	Inter-alpha-trypsin inhibitor H1−H4 chains
Carboxypeptidade B2	Isoform 3 of MAX gene-associated protein
Ceruloplasmin	Kininogen
Clusterin	Pigment ephitelium-derived factor
Coagulation factor V	Plasminogen
Coagulation factor XIII A chain	Pregnancy zone protein
Collagen alpha-2(I) chain	Proline-rich acidic protein 1
Collectin 11	Prothrombin
Complement C1q subcomponent subunit B	Serum albumin
Complement C1r subcomponent	Serum amyloid A-4 protein
Complement C1s subcomponent	Serum paraoxonase/arylesterase 1
Complement component C3	Tetranectin
Complement component C4-A	Transthyretin
Complement component C9	Vitamin K-dependent protein C
Complement factor H	Vitamin K-dependent protein S
Complement factor H-related protein	Vitronectin
Extracellular matrix protein 1	

## Data Availability

All mass spectrometry peptidomics data have been deposited to the ProteomeXchange Consortium (http://proteomecentral.proteomexchange.org), via the PRIDE partner repository [110] with the dataset identifier: PXD027997.

## Supplementary Materials

The following are available online at https://www.mdpi.com/article/10.3390/toxins13110764/s1, Supplementary Tables S1–S8: Peptides identified by LC-MS/MS analysis of the peptide fraction of human plasma after incubation with HF3. Supplementary Table S9: Peptides identified by LC-MS/MS analysis of the peptide fraction of human fibrinogen after incubation with HF3.
